# Relationship between Profile, Quality of Life and Burden of Older People With Dementia’s Caregivers during the COVID-19 Pandemic

**DOI:** 10.1007/s10823-026-09605-9

**Published:** 2026-11-03

**Authors:** Renata Carolina Gerassi, Larissa Pires de Andrade, Carolina Tsen, Maria Juana Beatriz Lima Candanedo, Jessica Bianca Aily, Ana Beatriz Simões Pereira, Grace Angélica de Oliveira Gomes, Juliana Hotta Ansai

**Affiliations:** 1 Department of Physical Therapy, Federal University of São Carlos, São Carlos, Brazil; 2 Department of Gerontology, Federal University of São Carlos, São Carlos, 13565-905 Brazil

**Keywords:** Aged, Dementia, Caregivers, Quality of Life, Caregiver burden

## Abstract

This study was to verify the quality of life and caregiver burden of older people with dementia during the Severe Acute Respiratory Syndrome (COVID-19) pandemic, and the clinical and sociodemographic factors associated with them. This cross-sectional study was conducted remotely and included Brazilian older people with a mild to moderate stage of dementia and their family caregivers. The caregivers reported older people’s clinical and sociodemographic characteristics. Caregiver characteristics were also collected. The sample comprised 37 pairs of older adults and caregivers, involving older adults with a mean age of 77.5 years old and caregivers who were mostly women (86.5%). Only caregiver years of study and caregiver burden significantly influenced caregiver quality of life. Caregiver distress related to older people’s behavior symptoms, older people’s behavioral symptoms and caregiver quality of life influenced caregiver burden. These caregivers have a fair quality of life and moderate to mild burden during the COVID-19 pandemic. The fewer years of study, the higher quality of life the caregiver presents. The lower caregiver quality of life, the higher caregiver distress related to behavioral symptoms, and the older people’s behavioral symptoms, the higher the caregiver burden is.

## Introduction

The emergence and incidence of severe acute respiratory syndrome (SARS-CoV-2) or COVID-19 presents new challenges for healthcare systems around the world. Numerous preventive measures, such as physical and social isolation, have been adopted to control the rate of this viral transmission. However, little is known about the consequences of these preventive measures for older people with dementia and their family caregivers [1].

The consequences of the COVID-19 pandemic in older adults include psychosocial stressors related to physical restrictions, communication barriers, lack of social support, a decrease in autonomy and prone to biological, psychological and social disorders [1, 2]. In older people with dementia, the pandemic has exacerbated symptoms such as anxiety, aggression, insomnia, irritability, vulnerability and a lack of independence [3].

As a result, there was probably an increase in caregiver burden and, consequently, an increase in the demand and care responsibilities of their caregivers and family members [4]. Caregivers of older people with dementia tend to express a naturally greater burden and lower quality of life when compared to caregivers of people with other illnesses [5]. Thus, it is important to understand quality of life and burden of family caregivers who care for older people with dementia during the COVID-19 pandemic, especially in developing countries where there is a need for developing and implementing more strategies for this population.

Caregiver burden and quality of life during the COVID-19 pandemic may vary according to working hours, supervision and direct assistance provided [6, 7]. Other data can influence caregiver burden and quality of life, as caregivers present high levels of stress, depression, anxiety, morbidity, fatigue, low quality of life and physical problems associated with care, and they can be characterized as “hidden secondary patients” [6, 7]. Understanding the clinical and sociodemographic factors associated with caregiver quality of life and burden can help to face the negative impacts caused by the restriction measures adopted during the COVID-19 pandemic [8]. In Spain and Italy, studies show the increased use of telehealth, during the pandemic, as a care tool for people with dementia [9]. Remote interventions have been used to identify risk factors, monitor and support caregivers of people with dementia [10].

There is a need for studies in different geographic areas and socioeconomic segments. Given the challenges observed, the present study aimed to verify the quality of life and burden in family caregivers of older people with dementia during the COVID-19 pandemic, and clinical and sociodemographic factors associated with them. Thus, it is expected that this study will contribute in an investigative way regarding aspects related to the impacts caused by the pandemic in the Brazilian population.

## Methods

### Study Design and Participants

This study was carried out completely remotely with Brazilian older people diagnosed with dementia and family caregivers who were recruited from health services and social centers, publications in newspapers, magazines, radio and social media and in the Research Laboratory´s database. Inclusion criteria were: older people who have been diagnosed with dementia at a mild or moderate stage, attested by a neurologist researcher using the DSM V-TR diagnostic criteria [11] and the Clinical Dementia Rating Scale (CDR) [12]; their family caregivers who spend most of the day with an older person with dementia at least four times a week; and any family member who lives with an older person and is familiar with cell phones and applications to enable the proposed assessments and interventions.

Exclusion criteria for this study were: older people who are not allowed to practice physical activities at home; a history of severe muscle injuries, motor deficit due to neuromuscular diseases (Multiple Sclerosis, Huntington´s disease, epilepsy and head trauma); any functional or sensory impairment that prevents the proposed assessments (limb plegia or paresis, significant tremor, severe and uncorrected audiovisual disorder); individuals with advanced dementia; any cardiovascular or infectious condition present in the list of absolute contraindications for the Physical Activity Readiness Medical Examination [13]; caregivers who do not have preserved cognition screened by the Mini Mental State Examination [14] and are unable to read orientations in the support materials; and a compromised Internet signal.

### Outcomes

The evaluations followed the recommendations contained in the Remote Administration Guidelines for the NIH Toolbox®: Response to COVID-19 [15]. The evaluations were performed by a previously trained researcher through video calls using Google Meet at a time that suited the caregiver and the older person. The evaluations of all the individual's assessment times during the research were performed in the same period (morning or afternoon) agreed upon initially by the same evaluator.

Initially, the caregiver responded to an anamnesis questionnaire with the older person’s clinical and sociodemographic characteristics, including age, gender, stage of dementia by the Clinical Dementia Rating—CDR [12], behavior symptoms by the Neuropsychiatric Inventory—NPI [16] and functional capacity by the World Health Organization Disability Assessment Schedule—WHODAS 2.0 [17].

Furthermore, the caregiver characteristics were collected, including age, gender, years of study, hours dedicated to care per day, general health and distress related to the older person’s behavior symptoms by the NPI [16].

Caregiver quality of life was assessed by the Quality-of-life scale for patients with Alzheimer's disease – caregiver version (QoL-AD) [18]. It is divided into 13 dimensions (physical health, energy, mood, living situation, memory, family, marriage, friends, self as a whole, ability to do chores around the house, ability to do things for fun, money, and life as a whole). Each domain presents response options that include 1(poor), 2(fair), 3(good) and 4 (excellent). The total score is 13–52 points, and higher scores indicates a better QoL [19].

The Zarit Burden Scale [20] was also applied to assess caregiver burden. This 22-item scale measures how caregiving impacts the caregiver's physical and emotional health, as well as his/her social activities and financial condition. All 22 items present a five-point scale that describes how each statement affects the person. The cutoff point for caregiver burden was the following score: intense overload scores between 61 and 88; moderate to severe between 41 and 60; moderate to mild between 21 and 40; and absence of overload scores below 21 points [21].

### Data Analysis

Quality of life and caregiver burden in family caregivers of older people with dementia during the COVID-19 pandemic were descriptively analyzed. A significance level of α = 0.05 was adopted and SPSS (22.0) software was used to perform the statistical tests. The Shapiro–wilk normality test was applied for all continuous variables to verify data distribution. The simple linear regression was applied to verify which clinical and sociodemographic factors influenced the caregiver's quality of life and burden.

## Results

The sample comprised 37 pairs of older adults and caregivers, involving older adults with a mean age of 77.5 years old, mostly female (68.4%), and caregivers who were mostly female (86.5%), with a mean age of 53.65 years old. The mean QoL-AD score was 25.9 points, which indicates fair caregiver quality of life. The mean Zarit Burden Scale score was 31.1 points, which means moderate to mild caregiver burden (Table 1).

**Table 1:** Clinical and sociodemographic data (n = 37)

Caregiver characteristics	m ± SD or n (%)
Age (years)	53.6 ± 10.30
Female gender	32 (86.5)
Years of study	14.5 ± 5.07
Hour dedicated to care per day	10.5 ± 9.02
General health	
Good	24 (64.9)
Very good	13 (35.1)
NPI (distress related to the older person’s behavior symptoms)	12.3 ± 8.41
Quality of life	25.9 ± 3.65
Caregiver burden	31.1 ± 10.75
**Older person with dementia characteristics**	**m ± SD or n (%)**
Age (years)	77.5 ± 6.48
Female gender	29 (68.4)
CDR	
1 (Mild)	25 (67.6)
2 (Moderate)	12 (32.4)
NPI (behavior symptoms)	28.4 ± 21.71
WHODAS 2.0	17.5 ± 8.97

m ± SD = mean ± Standard Deviation, n (%)=number (percentage), NPI = Neuropsychiatric Inventory, CDR = Clinical Dementia Rating, WHODAS = The World Health Organization Disability Assessment Schedule.

**Fig. 1:**
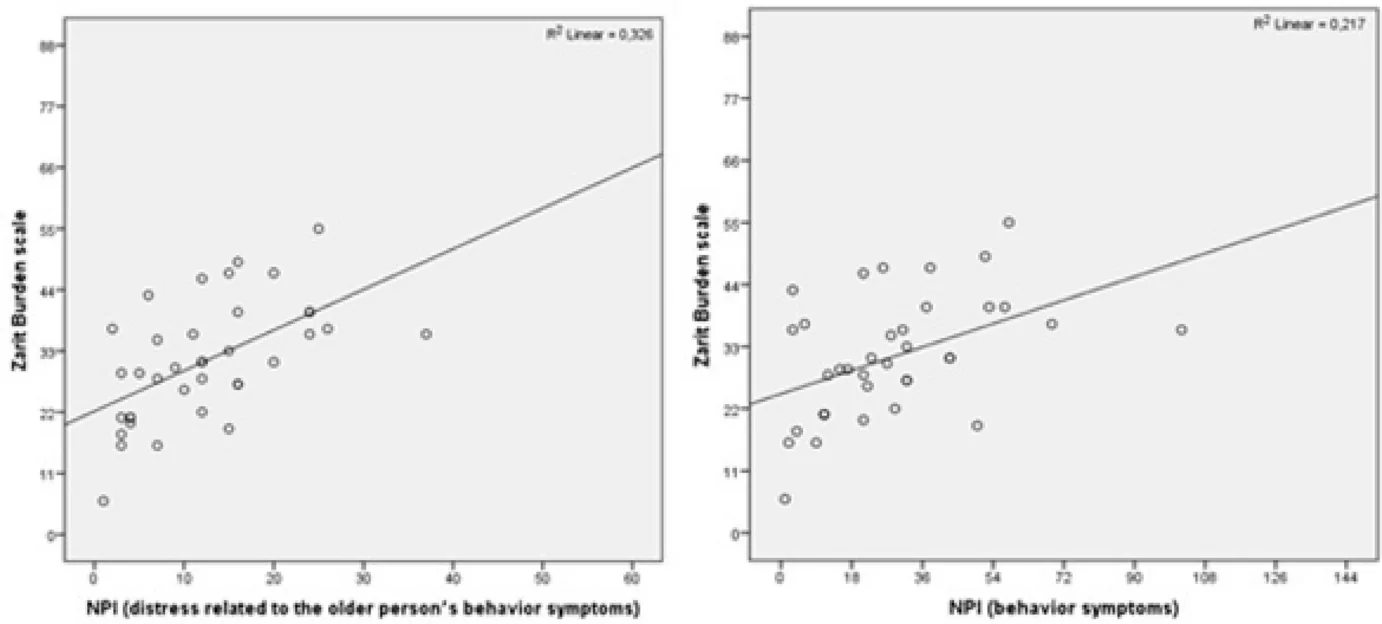
Simple linear representing the association between caregiver quality of life with caregiver years of study and caregiver burden

Regarding clinical and sociodemographic factors associated with quality of life in family caregivers of older people with dementia during the COVID-19 pandemic, only caregiver years of study and caregiver burden significantly influenced the quality of life. The less years of study, the higher the quality of life the caregiver presents, and the caregiver with more years of study presents a lower quality of life. Furthermore, the higher the caregiver burden, the lower the quality of life the caregiver presents, which can also show that the lower the caregiver burden, the higher the caregiver quality of life is (Table 2, Fig. 1).

**Table 2:** Clinical and sociodemographic factors associated with quality of life (n = 37)

Characteristics	B (95% CI)	R^2^	P value
Caregiver—age	0.087 (− 0.031–0.205)	0.060	0.143
Caregiver – gender	0.725 (− 2.885–4.335)	0.005	0.686
Caregiver—years of study	−0.287 (− 0.514–0.061)	0.159	**0.014**
Caregiver—hour dedicated to care per day	0.021 (− 0.117–0.160)	0.003	0.757
Caregiver—general health	0.042 (− 2.550–2.633)	0.000	0.074
Caregiver—NPI (distress related to the older person’s behavior symptoms)	−0.015 (− 0.164–0.134)	0.001	0.838
Caregiver burden	−0.157 (− 0.261-(− 0.054)	0.214	**0.004**
Older person with dementia—age	0.047 (− 0.146–0.239)	0.007	0.627
Older person with dementia—gender	−2.836 (− 5.680–0.007)	0.105	0.051
Older person with dementia—CDR	1.890 (− 0.672–4.452)	0.060	0.143
Older person with dementia—NPI (behavior symptoms)	0.010 (− 0.048–0.067)	0.003	0.732
Older person with dementia—WHODAS	0.107 (− 0.028–0.242)	0.069	0.117

N = number of individuals; CI = confidence interval, **p ≤ 0.05**, NPI = Neuropsychiatric Inventory, CDR = Clinical Dementia Rating, WHODAS = The World Health Organization Disability Assessment Schedule.

**Fig. 2:**
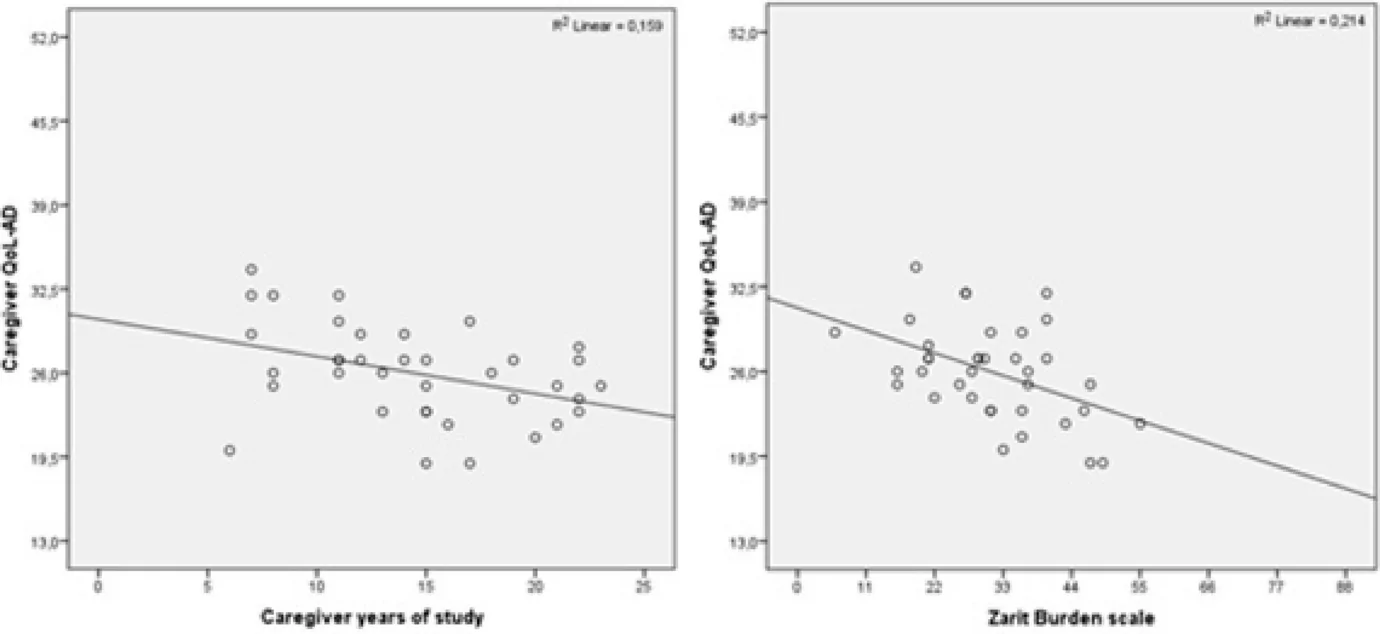
Simple linear representing the association between caregiver burden with distress related to the older person’s behavior symptoms and behavior symptoms

When considering caregiver burden, caregiver distress related to the older person’s behavior symptoms, older person’s behavioral symptoms, and caregiver quality of life significantly influenced the caregiver burden. They are also inversely proportional values, meaning that the higher the caregiver quality of life, the lower the caregiver burden is. The greater the caregiver distress related to behavioral symptoms and the older person’s behavioral symptoms, the greater the caregiver burden is. Moreover, the less caregiver distress related to behavioral symptoms and the older person’s behavioral symptoms, the less the caregiver burden is (Table 3, Fig. 2).

**Table 3:** Clinical and sociodemographic factors associated with caregiver burden (n = 37)

Characteristics	B (95% CI)	R^2^	P value
Caregiver—age	−0.131 (− 0.487–0.224)	0.016	0.458
Caregiver—gender	−1.144 (− 11.784–9.497)	0.001	0.829
Caregiver—years of study	−0.031 (− 0.758–0.696)	0.000	0.931
Caregiver—hour dedicated to care per day	0.383 (− 0.005–0.770)	0.103	0.053
Caregiver—general health	−6.340 (− 13.648–0.968)	0.081	0.087
Caregiver—NPI (distress related to the older person’s behavior symptoms)	0.730 (0.370–1.090)	0.326	**0.005**
Caregiver—quality of life	−1.361 (− 2.256-(− 0.466)	0.214	**0.004**
Older person with dementia—age	−0.162 (− 0.729–0.404)	0.010	0.564
Older person with dementia—gender	5.659 (− 2.967–14.286)	0.048	0.192
Older person with dementia—CDR	−0.897 (− 8.667–6.873)	0.002	0.816
Older person with dementia—NPI (behavior symptoms)	0.231 (0.080–0.381)	0.217	**0.004**
Older person with dementia—WHODAS	−0.102 (− 0.512–0.308)	0.007	0.617

N = number of individuals; CI = confidence interval, **p ≤ 0.05**, NPI = Neuropsychiatric Inventory, CDR = Clinical Dementia Rating, WHODAS = The World Health Organization Disability Assessment Schedule.

## Discussion

The aim of this study was to verify quality of life and caregiver burden in family caregivers of older people with dementia during the COVID-19 pandemic and clinical and sociodemographic factors associated with them. As the main findings, this study shows that caregivers presented a fair quality of life and moderate to mild burden. Moreover, caregiver years of study and caregiver burden significantly influenced caregiver quality of life; and, finally, caregiver distress related to older people’s behavior symptoms, older people’s behavioral symptoms and caregiver quality of life influenced caregiver burden.

According to a meta-analysis study [22], the incidence of dementia increases linearly with age at least until 90 years old and there is no difference between genders. However, women tend to have a higher incidence of Alzheimer's disease at a very advanced age, and men a higher incidence at younger ages, which justifies the prevalence of women and the mean age of older people with dementia in the present study. In our sample, most caregivers were women, with a mean age of 53.6 years old. It is well established that caregivers of older people with dementia are usually family members and most of them are women, even with huge cultural changes in recent decades [23, 24].

A moderate to mild caregiver burden was found in the present study. It is well known that caregivers of older people with dementia tend to express a naturally greater burden compared to caregivers of older adults without dementia [5, 25]. However, considering the COVID-19 pandemic and its consequences, the observed caregiver burden was even less than the hypothesized one. In the same way, caregiver quality of life was considered fair, even in the COVID-19 context. Both findings appear to be related to the high educational level of caregivers, resulting in better jobs, higher salaries, and more resources for health care, which may directly contribute to better caregiver quality of life and less burden [26].

Caregiver years of study was an important associated factor with their quality of life during the COVID-19 pandemic. In other words, the high educational level of caregivers in the present sample was surprisingly associated with a low caregiver’s quality of life. This finding is contrary to the previous literature, which showed that a lower educational level is associated with a worse state of general health [27]. However, this finding can be supported by the overwhelming manifestation and spread of COVID-19, which has resulted in the interruption of daily care activities for people with dementia [5], and consequently, increased family care. Highly educated caregivers tend to have more intellectual jobs, with huge responsibilities [26]. Although speculative, these responsibilities associated with family care may result in overburden and/or poor quality of life [26].

The caregiver burden was the factor that most influenced caregiver quality of life. Moreover, caregiver quality of life influenced caregiver burden. Similarly, a study conducted in Brazil found significant correlations between a lower level of caregiver burden and better perceptions of quality of life in family caregivers of frail older people. The authors also found an association between a higher degree of older people’s dependence and a higher caregiver burden and less caregiver quality of life [28].

In the present study, older people’s behavioral symptoms and mainly caregiver distress related to older people’s behavior symptoms also influenced caregiver burden. This finding corroborates with a systematic review, which aimed to determine whether known symptoms or symptom clusters are associated with a negative impact on caregiver depression and burden. Taking this into account, the authors concluded that depression, aggression and sleep disorders are the symptoms most often identified to negatively impact caregivers and their burden [29]. In addition, a recent study conducted during the COVID-19 pandemic noted that behavioral symptoms in older people appear to arise from social restrictions that occur as a consequence of the pandemic. Thus, implementing caregiver support and the presence of qualified professionals in nursing homes are necessary to restore social interaction and adjust technological support to patients´ needs [30].

The authors acknowledged limitations of this study, including the possible omission of data during telephone calls and video calls, the study design with absence of a cause-and-effect relationship and the low sample size. On the other hand, the assessments applied remotely may be useful to monitor and assist caregivers during the COVID-19 pandemic and to track the negative consequences of the pandemic in family caregivers of older adults with dementia. The present study brought new information about the factors associated with quality of life and burden of care in Brazilian family caregivers during the pandemic. Thus, healthcare providers must pay attention to these important clinical and sociodemographic characteristics while providing healthcare and specific strategies for family caregivers of older people with mild to moderate dementia. Studies involving the longitudinal design on family caregivers of older people with specific types of dementia are suggested to better understand the influence on clinical and sociodemographic factors with quality of life and caregiver burden during the pandemic and how to minimize its impacts.

In conclusion, family caregivers of older people with dementia presented a fair quality of life and moderate to mild burden during the COVID-19 pandemic. The fewer years of study, the higher quality of life the caregiver presents. The lower caregiver quality of life, the higher the caregiver distress related to behavioral symptoms and the older people’s behavioral symptoms, the higher the caregiver burden is.

Policy impact statement: This study has found that quality of life and burden of family caregivers of older adults with dementia during the COVID-19 pandemic are associated with clinical and sociodemographic factors. This suggests that policy strategies should be made to address these factors, as well as to establish telehealth programs for this population.

Practice impact statement: This study is an example of how clinical and sociodemographic factors might be related to quality of life and burden of family caregivers. Thus, healthcare professionals must pay attention to each of these factors while conducting the treatment.

## Data Availability

The data that support the findings of this study are available on request from the corresponding author. The data are not publicly available due to privacy or ethical restrictions.
